# Supplementary material for: Grief Iconography between Italians and Americans: A Comparative Study on How Mourning Is Visually Expressed on Social Media
Source: Behav Sci (Basel). 2021 Jul 20;11(7):104. doi: 10.3390/bs11070104 (PMC8301040; doi:10.3390/bs11070104)
Supplement: Supplementary file 1 [file behavsci-11-00104-s001.zip › behavsci-1180021-supplementary.pdf]

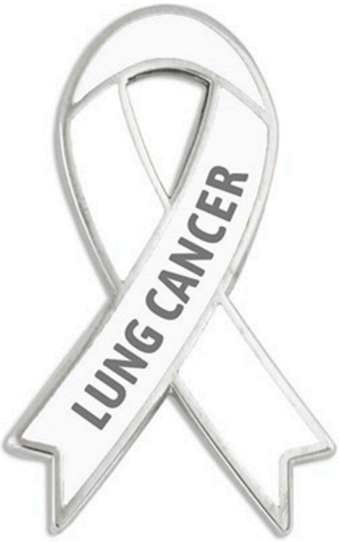

This representation has been coded as "image" and "object".

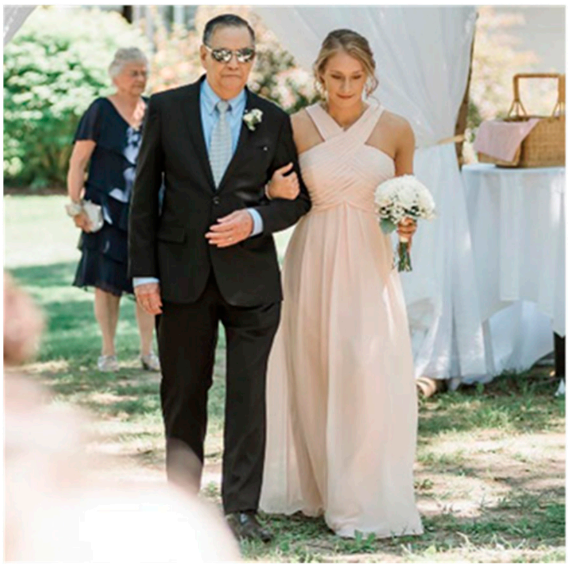

This representation has been coded as "picture", "person", "conjoint appearance" (father and daughter are depicted) and "special occasion" (a wedding is depicted).

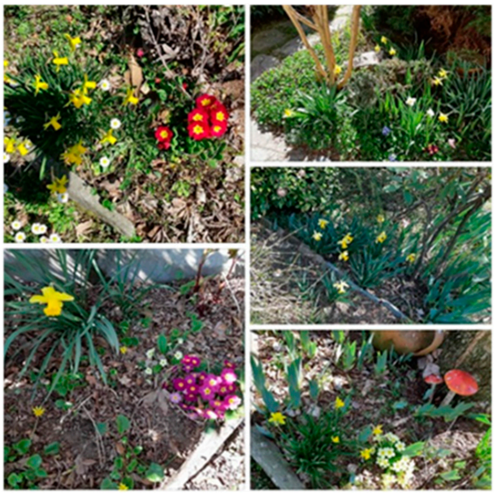

This representation has been coded as "picture", "object" and "refined representation".

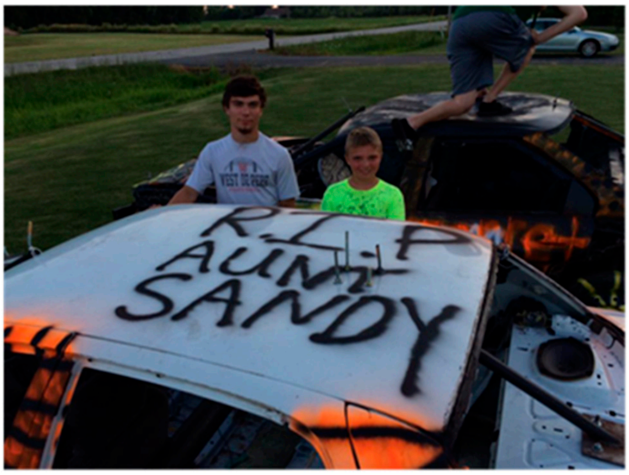

This representation has been coded as "image", "person", and "object" (we used both categories because both mourners and the car recieve some consideration).

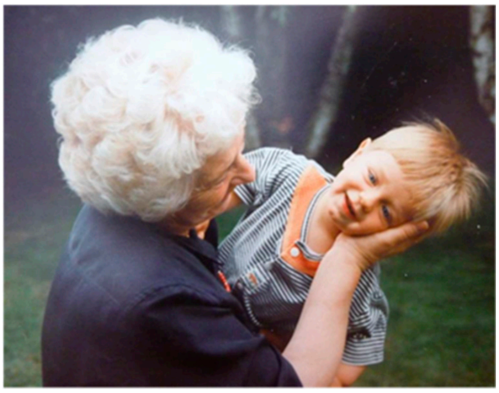

This representation has been coded as "picture", "person", "conjoint appeareance", "positive emotional connotation", "youth of the participant" and "act of kindess and caring".

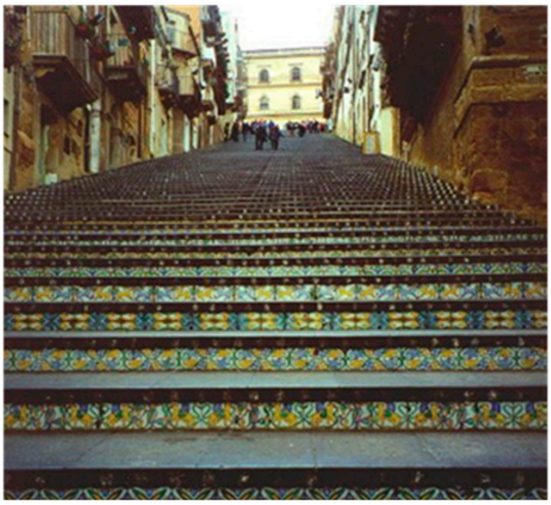

This representation has been coded as "picture" and "setting.

Figure S1. Six images sent by participants and coded by authors.
